# The Analysis of *Leontopodium leontopodioides* (Willd.) Beauv. Chemical Composition by GC/MS and UPLC-Q-Orbitrap MS

**DOI:** 10.1155/2024/3525212

**Published:** 2024-05-07

**Authors:** Yuanyuan Chen, Yu Dong, Lin Song, Changxi Bai, Buhechaolu Wang, Chula Sa

**Affiliations:** College of Mongolian Medicine, Inner Mongolia Medical University, Hohhot 010110, China

## Abstract

*Leontopodium leontopodioides* (Willd.) Beauv. (*L. leontopodioides*.) has been used to treat lung diseases in traditional Chinese medicine (TCM). However, a systematic analysis of its chemical components has not been reported so far. In this study, UPLC-Q-Orbitrap MS and GC-MS were applied to investigate the chemical composition of the water extracts and essential oils of *L. leontopodioides.* UPLC-Q-Orbitrap MS adopts a heating electrospray ionization source, collecting primary and secondary mass spectrometry data in positive and negative ions, respectively, and uses Compound Discoverer 3.2 software to analyze the collected raw data. As a result, a total of 39 compounds were identified from their high-resolution mass spectra in both positive and negative ionization modes, including 13 flavonoids and their glycosides, 15 phenolic acids, 4 oligosaccharides and glycosides, 4 pentacyclic triterpenoids, and 3 other compounds. Among them, 18 chemical components have not been reported in *L. leontopodioides*. In the GC-MS section, two common organic solvents (n-hexane and diethyl ether) were used to extract essential oils, and the mass spectra were recorded at 70 eV (electron impact) and scanned in the range of 35∼450 m/z. Compounds were identified using NIST (version 2017), and the peak area normalization method was used to calculate their relative amounts. Finally, 17 components were identified in the volatile oil extracted with n-hexane, accounting for 80.38% of the total volatile oil, including monoterpenoids, phenylpropene, fatty acids, and aliphatic hydrocarbons. In the volatile oil extracted with diethyl ether, 16 components were identified, accounting for 73.50% of the total volatile oil, including phenylpropene, aliphatic hydrocarbons, monoterpenoids, fatty acids, and esters. This study was the first to conduct a comprehensive analysis of the chemical composition of the *L. leontopodioides* water extract and its essential oil, and a comprehensive chemical composition spectrum was constructed, to lay a foundation for its further pharmacodynamic material basis and quality evaluation.

## 1. Introduction

**Figure 1:**
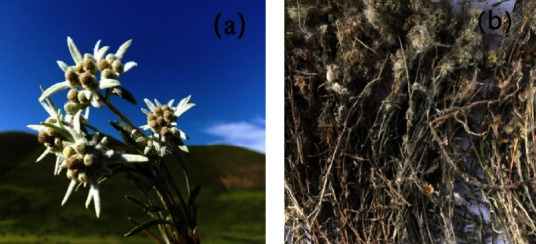
Plant diagram (a) and medicinal herbs diagram (b) of *L. leontopodioides*.

As a traditional Chinese medicine, *Leontopodium leontopodioides* (Willd.) Beauv. has the functions of clearing away the pulmonary heat, relieving cough, and expectorating phlegm and is generally used to treat lung diseases in TCM [1]. *L. leontopodioides* belongs to the Asteraceae family and is a perennial herb with a height of 5∼45 cm, and it is widely distributed in northeast, north, and northwest China and grows in arid grasslands, loess slopes, gravel, and mountain grasslands at an altitude of 100∼3200 m [2], as shown in Figure 1. Previous studies of *L. leontopodioides* were isolated by chromatographic methods, such as silica gel, ODS, Sephadex LH-20, and HPLC, and identified by chemical and physical methods, especially spectral analysis [3–5]. Modern pharmacological studies have shown that *L. leontopodioides* has anti-inflammatory, antibacterial, antioxidant, hypoglycemic, diuretic, and other effects [6–9]. Chen [10] et al. used chemical and spectroscopic methods to study a 70% EtOH extract of the whole plants of *Leontopodium leontopodioides* (Wild.) Beauv obtained leontoaerialosides A (1), B (2), C (3), D (4), and E (5). Zhao et al. [11] found that chlorogenic acid and ferulic acid are components with obvious antioxidant effects in *L. leontopodioides*, and most of the chemical components related to antioxidant activity are phenolic acids. Wu et al. [12] obtained an abundant higher monomer compound by silica gel column chromatography and preparative thin-layer chromatography in ethyl acetate parts of the alcohol extract; through analysis of ultraviolet, infrared, hydrogen, and carbon spectrum, it was presumed as para-hydroxyl-acetophenone. Gao et al. [13] reported the essential oil from the aerial parts of *L. leontopodioides* and found that it not only has low antioxidant activity but also possesses a potent antibacterial activity against *S. aureus* and *B. subtilis*. Although some chemical components, such as flavonoids, phenylpropanoids, phenolics [14], and essential oils, have been isolated from *L. leontopodioides*, systematic analysis of its chemical components has not been investigated. More importantly, *L. leontopodioides* has not been recorded in the Chinese Pharmacopoeia. Therefore, it is necessary to carry out a systematic and comprehensive study of the chemical composition in order to elucidate its pharmacodynamic material basis. At present, there is no research on the chemical composition of the water extract and essential oil of *L. leontopodioides* at the same time.

## 2. Experimental

### 2.1. Sample Preparation

The whole plant of *L. leontopodioides* was powdered. Pulverized samples of *L. leontopodioides* (50g) were accurately weighed, add 15 times of distilled water, decocting 3 times for 1 hour each time, combining the three filtrates, evaporating, drying and weighing to prepare the *L. leontopodioides* water extrac, and its yield was 27.63%. Fifty grams of crushed *L. leontopodioides* was precisely weighed, and the supercritical CO_2_ extraction method was used for 5 h at a temperature of 45°C and a pressure of 18 MPa to obtain 1.20 mL of dark green essential oil of *L. leontopodioides.*

An appropriate amount of *L. leontopodioides* water extract was weighed, and 1 mL of 80% methanol was added to prepare a solution with a concentration of 10 mg·mL^−1^, vortexed, ultrasonicated for 10 min, and centrifuged at 14000 rpm for 10 min. Then, 0.8 mL of the supernatant was placed in a centrifuge tube and centrifuged again, and the supernatant was placed into a sample bottle for analysis by UHPLC-MS.

### 2.2. Methods

In this study, water extracts and essential oils of *L. leontopodioides* were analyzed using UPLC-Q-Orbitrap MS and GC-MS techniques, respectively. Featuring high resolution, high sensitivity, and high speed, ultra-performance liquid chromatography quadrupole-Orbitrap mass spectrometry (UPLC-Q-Orbitrap MS), a cutting-edge molecular separation and determination technique, has been applied to the analysis of various complex samples [15, 16]. Based on UPLC-Q-Orbitrap MS technology, a rapid identification method was established for the chemical composition of the water extract of *L. leontopodioides*. According to the precise molecular mass and fragmentation information of the compounds, the main compounds were identified by means of databases and references, and their cracking laws were discussed, which provided a data basis for further elucidating their pharmacodynamic material basis. GC-MS a highly effective and versatile analytical technique is widely used in pharmaceutical industries for analytical research and development, quality control, and quality assurance [17]. The essential oil was extracted by supercritical carbon dioxide extraction and then dissolved in n-hexane and diethyl ether. The resulting fractions were analyzed by GC-MS, using NIST (version 2017) for similarity search, enabling identification of the components, while calculating their relative amounts using peak area normalization. The instruments and materials used in the experiment are listed in Table 1.

#### 2.2.1. Chromatography and Mass Spectrometry Conditions

The UPLC-Q-Orbitrap LC/MS system used a Waters ACQUITY UPLC HSS T3 C_18_ column (2.1 mm × 100 mm, 1.8 *μ*m; Waters Corporation, USA); the column temperature was 35°C, and the flow rate was 0.2 mL·min^−1^. The injection volume was 5 *μ*L; mobile phase was 0.1% formic acid acetonitrile (A)-0.1% formic acid water (B), gradient elution: 0∼10 min, 100% B; 10∼20 min, 100%∼70% B; 20∼25 min, 70%∼60% B; 25∼30 min, 60%∼50% B; 30∼40 min, 50%∼30% B; 40∼45 min, 30%∼0% B; 45∼60 min, 0% B; 60∼60.1 min, 0%∼100% B; and 60.1∼70 min, 100% B. The wavelength of DAD was set as a full scan in the range of 190∼400 nm.

The heating electrospray ionization source (HESI) was used as the ion source to detect the positive and negative ion modes, the positive spray voltage was 3.2 kV, the negative spray voltage was 3.0 kV, the detection method was full MS/dd-MS2, the sheath gas flow was 40 arb, the auxiliary gas flow rate was 15 arb, the capillary temperature was 320°C, and the auxiliary gas heater temperature was 350°C. The resolution of MS was 70000, the resolution of MS/MS was 17500, and the mass spectrum was recorded with a positive ion spectrum scan range of m/z 100∼500. Unknown compounds were identified using Compound Discoverer 3.2 software, and mzCloud (https://www.mzcloud.org/) and mzVault (self-built database) were used to identify compounds.

#### 2.2.2. GC-MS Conditions

Inject 1.0 *μ*L essential oils dissolved in n-hexane/diethyl ether each in splitless mode. An HP-5 ms fused silica capillary column (30 m, inner diameter 0.25 mm, and film thickness 0.25 *μ*m) was used with helium as the carrier gas, and the oven temperature was increased from 80°C to 90°C at a rate of 3°C/min (maintained for 2 min), 95°C to 140°C at a rate of 3°C/min, 155°C to 185°C at a rate of 2°C/min, and finally 195°C at a rate of 5°C/min (hold for 8 min). The total run time was 54.5 min, and the ion source temperature was set at 250°C. The GC interface temperature was 270°C. The mass spectra were recorded at 70 eV (EI) and were scanned in the range 35∼450 m/z. Compounds were identified using the NIST Chemistry WebBook (https://webbook.nist.gov).

## 3. Results

### 3.1. Compounds Confirmation of *L. leontopodioides* Water Extract by UPLC-Q-Orbitrap MS

**Figure 2:**
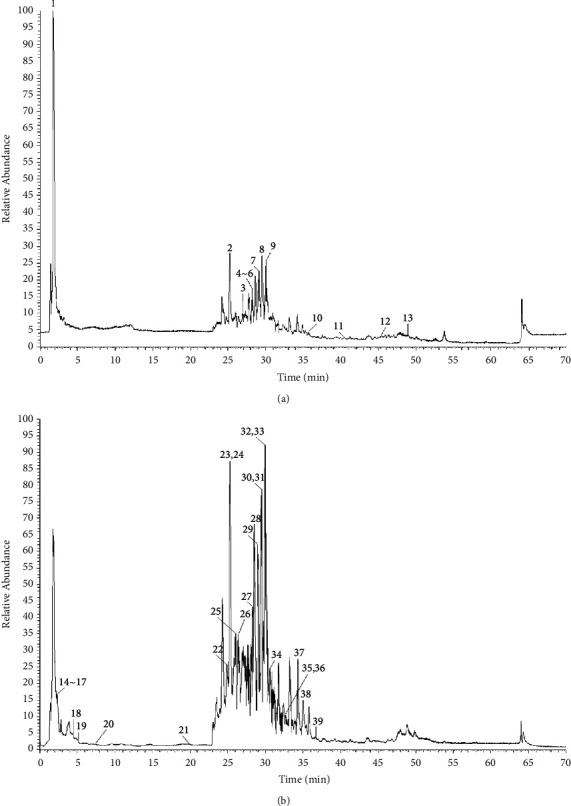
The total ion current (TIC) chromatograms of the water extract of *Leontopodium leontopodioides* (Willd.) Beauv. in positive (a) and negative (b) modes.

First, samples were injected according to the chromatographic and mass spectrometry conditions, and Compound Discoverer 3.2 software was used to search for the target compound peaks on the collected raw data and screen for compounds with a score greater than 80, and after matching, compounds were obtained, and the secondary fragmentation fragment ion information was analyzed to further accurately identify chemical components. As a result, a total of 39 chemical components were identified from the water extract of *L. leontopodioides*, mainly including flavonoids, phenolic acids, pentacyclic triterpenes, oligosaccharides, and glycosides. The total ion chromatogram is shown in Figure 2, and the chemical composition identification results are shown in Table 2.

#### 3.1.1. Identification of Flavonoids and Their Glycosides

**Figure 3:**
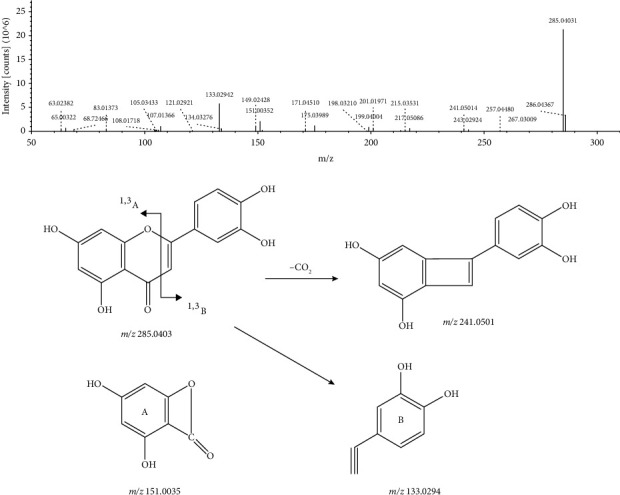
MS/MS spectrum and the fragmentation pathway of luteolin.

In this study, a total of 13 flavonoids and their glycosides (peaks 4, 5, 6, 7, 8, 9, 28, 31, 32, 35, 36, 37, and 38) were identified from the extract of *L. leontopodioides.* The excimer ion of peak 35 with a retention time of 32.827 min and a molecular formula of C_15_H_10_O_6_ given in negative ion mode was m/z 285.0403 [M − H]^−^, and it loses a molecule of carbon dioxide forming m/z 241.0501 [M − H − CO_2_]^−^. At the same time, 1, 3 cracking could occur to generate fragment ions m/z 151.0035 [M − H]^−^ and m/z 133.0294 [M − H]^−^. Among them, m/z 133.0294 consisted of residues on the B ring and C ring, and its intensity was larger than that of m/z 151.0035. Combined with the databases, peak 35 was identified as luteolin. Its MS/MS spectrum and the fragmentation pathway are shown in Figure 3.

**Figure 4:**
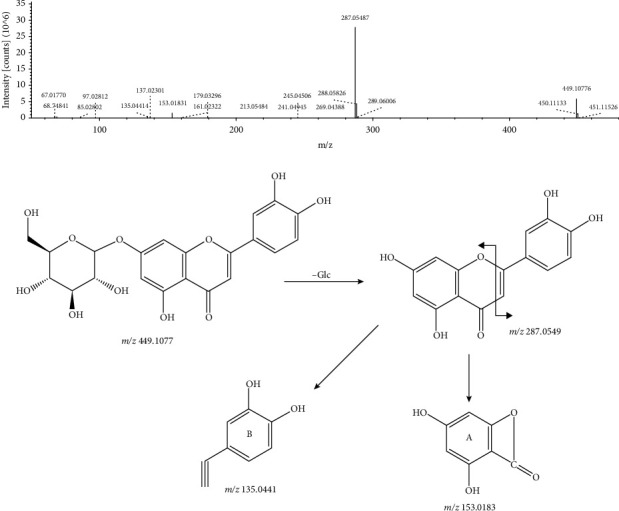
MS/MS spectrum and the fragmentation pathway of cynaroside.

Peak 9, with a retention time of 29.949 min and a molecular formula of C_21_H_20_O_11_, combined with the databases was identified as cynaroside. It responds well in positive ion mode, and the excimer ion given in positive ion mode was m/z 449.1077 [M + H]^+^. In secondary mass spectrometry, luteoloside lost a glucose to form aglycone ion m/z 287.0549 [M + H − Glc]^+^, and the aglycone was further cleaved by RDA to form fragment ions m/z 153.0183 [^1,3^A]^−^ and m/z 135.0441 [^1,3^B]^−^. Its MS/MS spectrum and the fragmentation pathway are shown in Figure 4.

**Figure 5:**
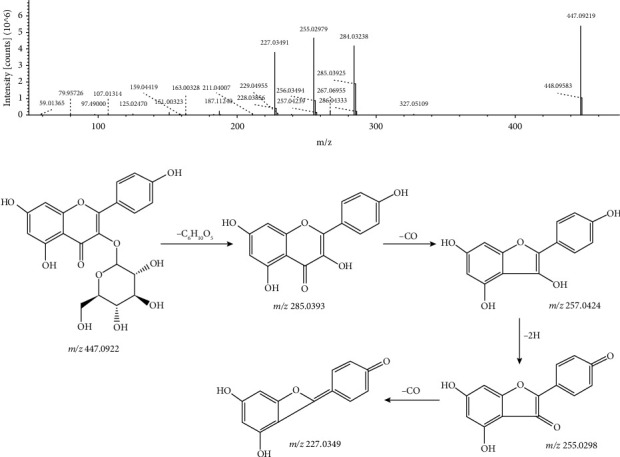
MS/MS spectrum and the fragmentation pathway of astragalin.

The excimer ion of peak 31 in negative ion mode was m/z 447.0922 [M − H]^−^. The excimer ion peaks were cracked and lost the fragment groups of C_6_H_10_O_5_ and C_6_H_11_O_5_, respectively, and fragment ions of m/z 285.0393 [M − H − C_6_H_10_O_5_]^−^ and m/z 284.0324 [M − H − C_6_H_11_O_5_]^−^ were obtained, respectively. Subsequently, the fragment ion of m/z 284.0324 continued to fragment, losing 1 neutral CO molecule, and producing a fragment ion of m/z 257.0424 [M − H − C_6_H_11_O_5_ − CO]^−^. At the same time, the fragment ion of m/z 285.0393 can continue to be fragmented, and after losing one neutral CO molecule, it rearranges and removes 2 H atoms, and a fragment ion of m/z 255.0298 [M − H − C_6_H_11_O_5_ − CO − 2H]^−^ was produced. Finally, the fragment ion continues to fragment and loses the CO molecule, producing a fragment ion of m/z 227.0349 [M − H − C_6_H_11_O_5_ − CO − 2H − CO]^−^. Compared with the databases, peak 31 was identified as astragalin. Its MS/MS spectrum and the fragmentation pathway are shown in Figure 5.

#### 3.1.2. Identification of Phenolic Acids

**Figure 6:**
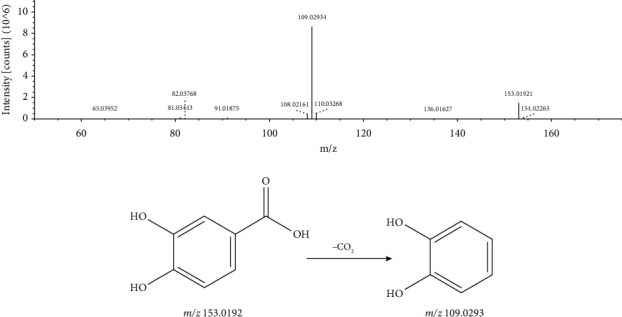
MS/MS spectrum and the fragmentation pathway of protocatechuic acid.

A total of 15 phenolic acid compounds (peaks 2, 3, 14, 17, 18, 21, 22, 23, 24, 26, 27, 29, 30, 33, and 34) were detected in the extract of *L. leontopodioides*, and these compounds responded better in negative ion mode. The excimer ion of peak 21 with a retention time of 19.948 min and a molecular formula of C_7_H_6_O_4_ in negative ion mode was m/z 153.0192 [M − H]^−^. Through the loss of CO_2_, the secondary spectrum generates fragment ion peaks at m/z 109.0293 [M − H − CO_2_]^−^. Compared with the databases, it was identified as protocatechuic acid. Its MS/MS spectrum and the fragmentation pathway are shown in Figure 6.

**Figure 7:**
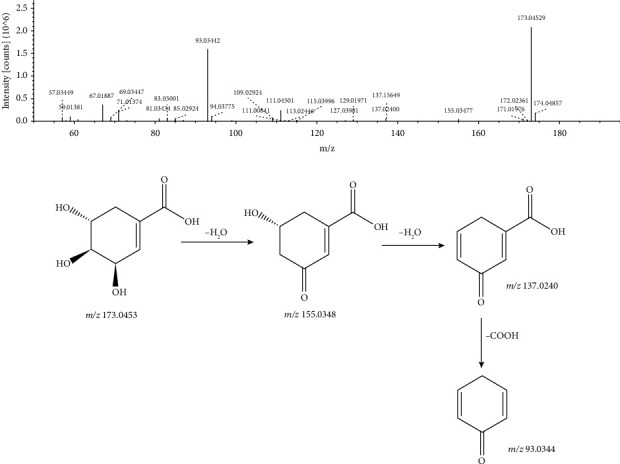
MS/MS spectrum and the fragmentation pathway of shikimic acid.

The excimer ion of peak 24 with a retention time of 25.764 min and a molecular formula of C_7_H_10_O_5_ in negative ion mode was m/z 173.0452 [M − H]^−^. The excimer ion peak lost one molecule of H_2_O to generate m/z 155.0348 [M − H − H_2_O]^−^ and also lost one molecule of H_2_O to generate m/z 137.0240 [M − H − 2H_2_O]^−^. Finally, this fragment ion continues to fragment and loses the COOH molecule, producing a fragment ion of m/z 93.0344 [M − H − 2H_2_O − COOH]^−^. Compared with the databases, peak 24 was identified as shikimic acid. Its MS/MS spectrum and the fragmentation pathway are shown in Figure 7.

#### 3.1.3. Identification of Pentacyclic Triterpenoids

**Figure 8:**
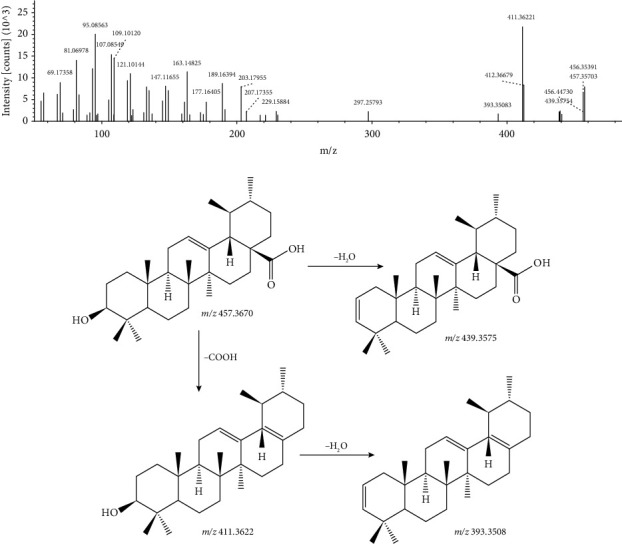
MS/MS spectrum and the fragmentation pathway of ursolic acid.

A total of four pentacyclic triterpenoids (peaks 10, 12, 13, and 39) were identified in this study. Ursolic acid is a pentacyclic triterpenoid with a retention time of 48.962 min and a molecular formula of C_30_H_48_O_3_. The excimer ion given in the positive ion mode was m/z 457.3670 [M + H]^+^. After it lost the neutral molecule H_2_O, a fragment ion of m/z 439.3575 was generated, and at the same time, the COOH molecule was lost to obtain a fragment ion of m/z 411.3622, and this fragment further lost H_2_O to produce the fragment ion of m/z 393.3508. Based on a comprehensive database, this structure was speculated as ursolic acid, and its MS/MS spectrum and the fragmentation pathway are shown in Figure 8.

#### 3.1.4. Identification of Oligosaccharides and Glycosides

**Figure 9:**
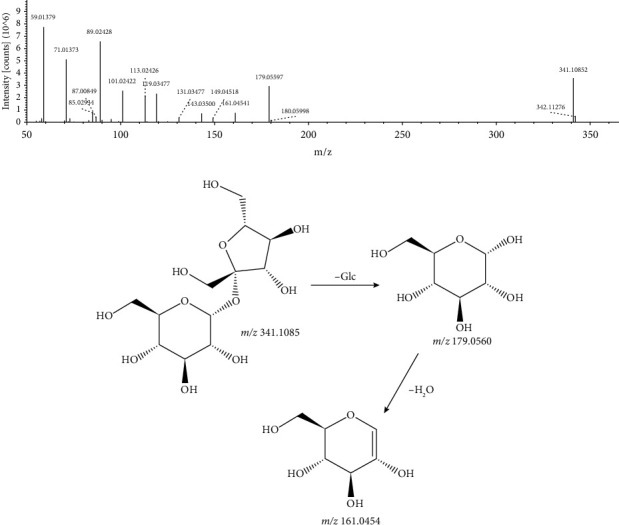
MS/MS spectrum and the fragmentation pathway of sucrose.

In the negative ion mode, the primary mass spectrometry mainly exists in the form of quasimolecular ion peak [M − H]^−^. Under the high-energy collision of mass spectrometry, the cleavage of the glycosidic bond mainly occurs and loses the glycosyl group. A total of four oligosaccharide and glycoside compounds (oligosaccharides: peaks 15, 16, and 19; glycosides: peak 20) were identified in this experiment. The excimer ion of peak 15 with a retention time of 2.189 min and a molecular formula of C_12_H_22_O_11_ in negative ion mode was m/z 341.1085 [M − H]^−^. Its negative ion mode of MS^2^ spectra revealed 179.0560 [M − H − Glc] and 161.0454 [M − H − Glc − H_2_O]. Based on a comprehensive database, it was tentatively identified as sucrose, and its MS/MS spectrum and the fragmentation pathway are shown in Figure 9.

#### 3.1.5. Other Compounds

**Figure 10:**
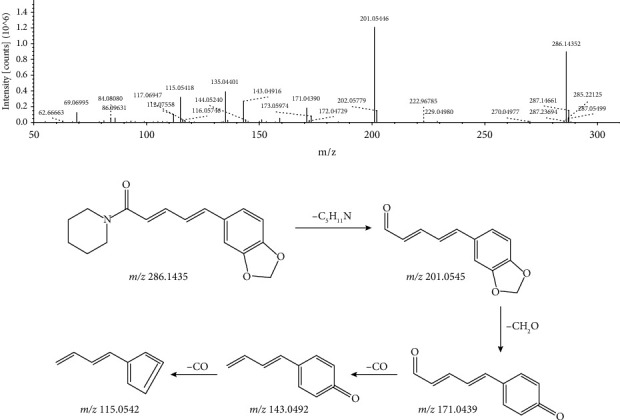
MS/MS spectrum and the fragmentation pathway of piperine.

In addition, two alkaloids (peaks 1 and 11) and one coumarin (peak 25) were identified in positive ion mode from the water extract of *L. leontopodioides*. The excimer ion of peak 11 with a retention time of 40.018 min and a molecular formula of C_17_H_19_NO_3_ in positive ion mode was m/z 286.1435 [M + H]^+^. The most abundant fragment of m/z 201.0545 was formed by the cleavage of the amide bond, and loss of piperidine ring (–C_5_H_11_N), in the process of further cracking, will be obtained the characteristic ion m/z 171.0439 [M + H − C_5_H_11_N − CH_2_O]^+^ with molecular formula C_11_H_7_O_2_. This ion continues to lose CO to obtain the ion m/z 143.0492 [M + H − C_5_H_11_N − CH_2_O − CO]^+^ and m/z 115.0542 [M + H − C_5_H_11_N − CH_2_O − CO − CO]^+^. Compared with the database, this compound was identified as piperine. Its MS/MS spectrum and the fragmentation pathway are shown in Figure 10.

### 3.2. Compound Confirmation of *L. leontopodioides* Essential Oil by GC-MS

**Figure 11:**
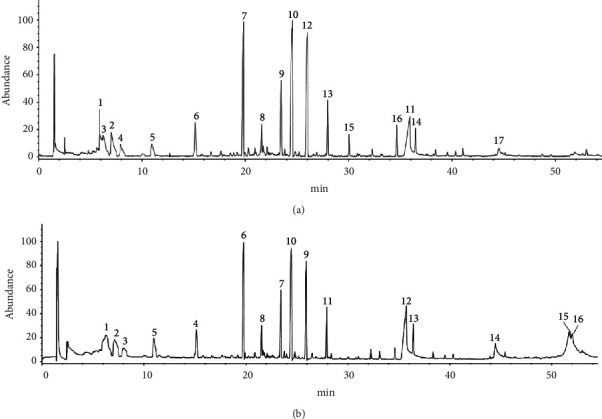
Total ion chromatogram of *n*-hexane (a)/diethyl ether and (b) extraction of *L. leontopodioides* essential oil [peaks 1, 2, 5, 8, 10, 11, and 14 are the same in (a) and (b)].

Samples were injected according to the GC-MS conditions and obtained a total ion chromatogram of volatile components in *L. leontopodioides*, and the obtained data were searched and matched by the mass spectrometry database of the National Institute of Standards and Technology (NIST 2017), and compounds with similarity scores above 80% were taken into account. After comparing the chemical composition of essential oils extracted with n-hexane and diethyl ether, a total of 33 volatile compounds (there were seven identical components) were identified, as shown in Figure 11 and Tables 3 and 4. The relative content of each component was estimated by the peak area normalization method. It can be seen from Table 3 that the main components of the volatiles of *L. leontopodioides* extracted with n-hexane mainly included phenylpropene (64.52%), monoterpenes (10.96%), fatty acids (10.03%), and contained some aliphatic hydrocarbons. Among them, the components with higher content were methylconiferylaldehyde (14.77%), (E)-2,6-dimethoxy-4-(prop-1-en-1-yl) phenol (12.04%), and eugenol (11.51%). As shown in Table 4, the main components included fatty acids (28.99%), phenylpropene (28.37%), aliphatic hydrocarbons, and some esters. Among them, the components with higher content were methylconiferylaldehyde (9.61%), pentadecanoic acid (9.25%), and 8-methylnonanoic acid (8.63%). Furthermore, terpinolene, terpinen-4-ol, *γ*-terpinene, methyleugenol, methylconiferylaldehyde, tetradecanoic acid, and n-hexadecanoic acid were identical components.

## 4. Discussion

### 4.1. UPLC-Q-Orbitrap MS Section

Flavonoids mainly exist in natural plants in the form of free or combined with sugar to form glycosides or in the form of carbon sugars, and they have anti-inflammatory, antioxidant, antibacterial, antidiabetic, antihypertensive, and other pharmacological activities [18]. The mass spectrometry fragmentation characteristics of flavonoid aglycones were mainly the loss of CO, COO, and CH_3_ groups, or the loss of neutral molecules such as H_2_O and the occurrence of reverse Diels–Alder reaction (RDA) fragmentation to form a series of characteristic ion peaks. Flavonoid glycosides first lose the glycosyl group to form the corresponding aglycone and then further cleave [19, 20]. In this study, taking luteolin, cynaroside, and astragalin as examples, the cracking rules of flavonoids and their glycosides were described, and it was found that the cracking rules of the three were consistent with those reported in the literature [21–23]. Among the 13 flavonoids and their glycosides obtained from the analysis, isoquercitrin, kaempferol, kaempferol-7-O-*β*-D-glucopyranoside, cymaroside, hyperoside, apigenin-7-O-*β*-D-glucoside, luteolin, quercetin, and apigenin compounds with previous reports [24–28] on the chemical composition of *L. leontopodioides*, scutellarein, morin, diosmetin, and astragalin have not been reported.

Phenolic acids mainly contain carbonyl, carboxyl, and hydroxyl groups, so neutral fragments of CO, H_2_O, and CO_2_ were easily lost in mass spectrometry collisions. The cracking rules of protocatechuic acid and shikimic acid obtained by database analysis are consistent with those reported in the literature [29, 30]. Chlorogenic acid, quinic acid, protocatechuic acid, protocatechualdehyde, cryptochlorogenic acid, caffeic acid, p-coumaric acid, isochlorogenic acid B, isochlorogenic acid C, and salicylic acid have been reported [31, 32]. Moreover, basic research on pharmacodynamics found that protocatechuic acid, protocatechuic aldehyde, chlorogenic acid, and caffeic acid in *L. leontopodioides* can resist acute inflammation [33]. Cinnamic acid, citric acid, fumaric acid, and shikimic acid have not been reported in *L. leontopodioides* in previous research.

The mass spectrometry fragmentation of pentacyclic triterpenoids was mainly loss of neutral molecules, such as H_2_O and CO, and the occurrence of Diels–Alder reaction, and the oligosaccharide and glycoside mass spectrometry was relatively simple. The cracking rules of ursolic acid and sucrose were consistent with literature reports [34, 35]. In previous research, pentacyclic triterpenoids, oligosaccharides, and glycosides have not been reported in *L. leontopodioides.* Newly discovered pentacyclic triterpenoids have a wide range of pharmacological effects and important biological activities, including anti-inflammatory, antibacterial, antiviral, immunomodulatory, blood sugar regulation, blood pressure lowering, and antitumor activities [36]. In particular, ursolic acid has the same inhibitory effect on glycosidase in vivo and in vitro and has an obvious hypoglycemic effect [37]. Oligosaccharides possess various bio-activities, including immune regulation, antitumor, antioxidation, and anti-infection, and modulate the gut microflora [38].

In addition, there has been no research on the chemical composition of *L. leontopodioides* using UPLC-Q-Orbitrap MS technology at present. UPLC-Q-Orbitrap MS technology adopts full MS/dd-MS2 mode, which greatly shortens the analysis time and can quickly detect multiple chemical components, with its advantages of high separation, high resolution, and high sensitivity, and it can provide accurate mass, elemental composition, mass spectrometry fragments, and other information required for the structural characterization of compounds without the need for reference substances. Then, the possible structure of the compound can be speculated for rapid qualitative analysis. This study collects data in both positive and negative ion modes to obtain more complete mass spectrometry data. Therefore, this method was used to analyze the water extract of *L. leontopodioides* in this study. Compared with previous studies, not only flavonoids and phenolic acids, but also pentacyclic triterpenes, oligosaccharides, and glycosides, which have never been reported before, were obtained using UPLC-Q-Orbitrap MS. The above research results show that the anti-inflammatory, antibacterial, antioxidant, and hypoglycemic effects of *L. leontopodioides* may be derived from the presence of chemical components, such as flavonoids, phenolic acids, pentacyclic triterpenes, oligosaccharides, and glycosides.

### 4.2. GC-MS Section

The essential oil of *L. leontopodioides* was extracted by supercritical carbon dioxide (SC-CO_2_) extraction technology. As a new advanced “green” separation technology, SC-CO_2_ is easy to operate and can not only extract and separate the desired substances quickly and efficiently but also the yield and purity of the obtained substances are higher than those of traditional methods [39].

The composition of *L. leontopodioides* essential oil was analyzed by GC-MS, and the chemical composition of essential oils extracted with different organic solvents was compared, and it was found that extracting essential oils with n-hexane can obtain a large amount of phenylpropene compounds, such as eugenol. The pharmacological effects of eugenol include antibacterial, anticancer, antioxidant, and other effects [40]; using diethyl ether to extract essential oils can obtain a large amount of fatty acids. Aparna et al. [41] through research suggested that the *n*-hexadecanoic acid might function as an anti-inflammatory agent. Analysis of *L. leontopodioides* essential oil by GC-MS found that the high content of fatty acid components and phenylpropene components may be an essential ingredient for its medicinal effect.

Gao et al. [13] extracted essential oil from aerial parts of *Leontopodium leontopodioides* (Willd.) Beauv. by water distillation and analyzed it by GC-FID and GC-MS. The main components in the essential oil were identified as palmitic acid (11.6%), *n*-pentadecanal (5.7%), linalool (3.8%), *β*-ionone (3.3%), hexahydrofarnesyl acetone (3.2%), bisabolone (3.2%), and *β*-caryophyllene (3.2%).

Compared with this, the results of this study are quite different, but the composition types are roughly the same, which may be related to the origin of *L. leontopodioides*, the extraction methods of volatile oil, and the extraction of volatile oil with different solvents.

## 5. Conclusions

In this study, UPLC-Q-Orbitrap MS and GC-MS analytical methods were established to comprehensively characterize the chemical composition of *L. leontopodioides* and provide a good research basis for the formulation compatibility and pharmacological mechanism of *L. leontopodioides*. However, the analysis of this study mainly focused on the identification and analysis of chemical components and did not carry out basic research on blood components and pharmacodynamic substances. Therefore, in the future, this analytical technique should be used to further improve the pharmacodynamic material basis of *L. leontopodioides*. At the same time, the mechanism of action of *L. leontopodioides* should be further elucidated by combining serum medicinal chemistry, network pharmacology, metabolomics, and other technologies.

## Figures and Tables

**Table 1:** Instruments and materials.

Instruments/materials	Type	Company
Ultra-high-performance liquid chromatography	Ultimate 3000	Dionex, USA
High-resolution mass spectrometer	Thermo Q Exactive Plus	Thermo Fisher Scientific, USA
Compound analysis and identification software	Compound Discoverer, 3.2	Thermo Fisher Scientific, USA
Gas/liquid chromatography-triple quadrupole-tandem mass spectrometer	EXPEC 5250	Hangzhou Puyu Technology Development Co., Ltd
Supercritical extraction equipment	GKSFE220-50-6L	Jiangsu Hi-Tech Pharmaceutical Equipment Co., Ltd
*n*-Hexane	Analytical grade	Tianjin Fuyu Fine Chemical Co., Ltd
Diethyl ether	Analytical grade	Tianjin Fuyu Fine Chemical Co., Ltd
Methanol	Mass spectrometry grade	Fisher Scientific (Pittsburg, PA, USA)
Acetonitrile	Mass spectrometry grade	Fisher Scientific (Pittsburg, PA, USA)
Formic acid	Mass spectrometry grade	Thermo Fisher Scientific, CN
Ultrapure water	—	—
*L. leontopodioides*	Whole plant (flowering period) of compositae *Leontopodium leontopodioides*	Xiaojinggou, Daqingshan, Inner Mongolia

**Table 2:** Compounds identified in *L. leontopodioides* water extract by UPLC-Q-Orbitrap MS.

No.	RT (min)	Formula	Ion type	MS	MS/MS fragment ions	PPM	Identification	Classification
1	1.773	C_5_H_11_NO_2_	[M + H]^+^	118.0861	103.0627 69.4371 59.0733	−1.20	Betaine^*∗*^	Alkaloids
2	25.258	C_16_H_18_O_9_	[M + H]^+^	355.1018	177.0533 163.0389 145.0286 135.0441	−1.72	Chlorogenic acid	Phenolic acids
3	26.935	C_9_H_8_O_2_	[M + H]^+^	149.0597	135.0443 121.0284 106.0414 89.0386	−0.40	Cinnamic acid^*∗*^	Phenolic acids
4	28.192	C_15_H_10_O_6_	[M + H]^+^	287.0544	269.0443 245.0444 223.0385 185.0599	−1.57	Scutellarein^*∗*^	Flavonoids
5	28.209	C_21_H_20_O_12_	[M + H]^+^	465.1023	303.0498 137.0235	−0.99	Isoquercitrin	Flavonoid glycosides
6	28.209	C_15_H_10_O_7_	[M + H]^+^	303.0495	285.0392 257.0443 229.0495 153.0183 137.0235	−1.62	Morin^*∗*^	Flavonoids
7	29.127	C_15_H_10_O_6_	[M + H]^+^	287.0545	259.0600 231.0648 153.0182	−1.57	Kaempferol	Flavonoids
8	29.448	C_21_H_20_O_11_	[M + H]^+^	449.1072	287.0548 153.0183 135.0441	−1.47	Kaempferol-7-O-*β*-D-glucopyranoside	Flavonoid glycosides
9	29.949	C_21_H_20_O_11_	[M + H]^+^	449.1077	287.0549 153.0183 135.0441	−1.47	Cynaroside	Flavonoid glycosides
10	36.069	C_42_H_60_O_16_	[M + H]^+^	823.4106	471.3467 453.3362 435.3276 285.2217 189.1639	−0.21	Dipotassium glycyrrhizinate^*∗*^	Pentacyclic triterpenoids
11	40.018	C_17_H_19_NO_3_	[M + H]^+^	286.1435	201.0545 171.0439 143.0492 115.0542	−1.50	Piperine^*∗*^	Alkaloids
12	45.320	C_30_H_46_O_3_	[M + H]^+^	455.3516	437.3575 409.3459 247.1696 201.0545 159.1167	−0.63	Oleanonic acid^*∗*^	Pentacyclic triterpenoids
13	48.962	C_30_H_48_O_3_	[M + H]^+^	457.3673	439.3575 411.3622 393.3508	−0.82	Ursolic acid^*∗*^	Pentacyclic triterpenoids
14	2.169	C_7_H_12_O_6_	[M − H]^−^	191.0560	147.0299 127.0400 109.0293 93.0344	−0.58	Quinic acid	Phenolic acids
15	2.189	C_12_H_22_O_11_	[M − H]^−^	341.1085	179.0560 161.0454	−0.86	Sucrose^*∗*^	Oligosaccharides
16	2.291	C_18_H_32_O_16_	[M − H]^−^	503.1619	341.1079 179.0556 161.0453 89.0243	−1.75	Raffinose^*∗*^	Oligosaccharides
17	2.421	C_6_H_8_O_7_	[M − H]^−^	191.0197	173.0085 154.9983 129.0194 111.0087 87.0086	0.02	Citric acid^*∗*^	Phenolic acids
18	4.794	C_4_H_4_O_4_	[M − H]^−^	115.0035	99.0087 73.0294	−1.75	Fumaric acid^*∗*^	Phenolic acids
19	5.439	C_24_H_42_O_21_	[M − H]^−^	665.2143	485.1498 179.0560 161.0452	−1.75	Nystose^*∗*^	Oligosaccharides
20	7.307	C_12_H_16_O_7_	[M − H]^−^	271.0823	161.0452 108.0216 71.0138	−0.21	Arbutin^*∗*^	Glycosides
21	19.948	C_7_H_6_O_4_	[M − H]^−^	153.0192	109.0293	−0.79	Protocatechuic acid	Phenolic acids
22	24.923	C_7_H_6_O_3_	[M − H]^−^	137.0243	119.0138 109.0294 91.0187	−1.24	Protocatechualdehyde	Phenolic acids
23	25.735	C_16_H_18_O_9_	[M − H]^−^	353.0874	191.0561 179.0349 135.0451	−1.14	Cryptochlorogenic acid	Phenolic acids
24	25.764	C_7_H_10_O_5_	[M − H]^−^	173.0452	155.0348 137.0240 93.0344	−2.54	Shikimic acid^*∗*^	Phenolic acids
25	26.388	C_9_H_6_O_4_	[M − H]^−^	177.0192	149.0243 133.0294 105.0345	−0.85	Esculetin^*∗*^	Coumarin
26	26.444	C_9_H_8_O_4_	[M − H]^−^	179.0349	135.0451	−0.59	Caffeic acid	Phenolic acids
27	28.156	C_9_H_8_O_3_	[M − H]^−^	163.0400	119.0501 93.0344	−0.73	p-coumaric acid	Phenolic acids
28	28.482	C_21_H_20_O_12_	[M − H]^−^	463.0880	300.0273 271.0247 243.0296	−0.34	Hyperoside	Flavonoid glycosides
29	28.912	C_25_H_24_O_12_	[M − H]^−^	515.1190	354.0908 336.0805 191.0560 179.0348 173.0453	−1.14	Isochlorogenic acid B	Phenolic acids
30	29.374	C_25_H_24_O_12_	[M − H]^−^	515.1190	353.0877 191.0561 179.0349 135.0452	−1.17	3,5-dicaffeoylquinic acid	Phenolic acids
31	29.437	C_21_H_20_O_11_	[M − H]^−^	447.0922	285.0393 284.0324 255.0298 227.0349	−0.53	Astragalin^*∗*^	Flavonoid glycosides
32	29.709	C_21_H_20_O_10_	[M − H]^−^	431.0981	269.0439 268.0375 153.0191	−0.73	Apigenin-7-O-*β*-D-glucoside	Flavonoid glycosides
33	29.852	C_25_H_24_O_12_	[M − H]^−^	515.1190	353.0876 335.0768 191.0560 179.0349 173.0453	−1.15	Isochlorogenic acid C	Phenolic acids
34	30.625	C_7_H_6_O_3_	[M − H]^−^	137.0243	94.0378 93.0344	−1.24	Salicylic acid	Phenolic acids
35	32.827	C_15_H_10_O_6_	[M − H]^−^	285.0403	241.0501 151.0035 133.0294	−0.83	Luteolin	Flavonoids
36	32.888	C_15_H_10_O_7_	[M − H]^−^	301.0351	273.0403 178.9985 151.0035 121.0293	−1.03	Quercetin	Flavonoids
37	34.706	C_15_H_10_O_5_	[M − H]^−^	269.0452	227.0351 225.0556 151.0035 117.0345	−1.30	Apigenin	Flavonoids
38	35.102	C_16_H_12_O_6_	[M − H]^−^	299.0559	284.0325 256.0376 227.0349 151.0034	−0.68	Diosmetin^*∗*^	Flavonoids
39	36.597	C_42_H_62_O_16_	[M − H]^−^	821.3963	351.0562 193.0346 113.0243 85.0294	−0.25	Diammonium glycyrrhizinate	Pentacyclic triterpenoids

^*∗*^Components that have not been reported in *L. leontopodioides*.

**Table 3:** Chemical composition of *L. leontopodioides* essential oil extracted with *n*-hexane.

No.	*t*_*R*_ (min)	Content (%)	Components	Formula	Relative molecular mass	Classification
1	5.91	2.61	Terpinolene^*∗*^	C_10_H_16_	136	Monoterpenoids
2	7.03	3.85	*γ*-Terpinene	C_10_H_16_	136	Monoterpenoids
3	6.23	2.56	Isoterpinolene	C_10_H_16_	136	Monoterpenoids
4	7.91	2.55	2-Carene^*∗*^	C_10_H_16_	136	Aliphatic hydrocarbons
5	10.94	1.94	Terpinen-4-ol	C_10_H_18_O	154	Monoterpenoids
6	15.14	3.24	3-Methoxycinnamaldehyde^*∗*^	C_10_H_10_O_2_	162	Phenylpropene
7	19.83	11.51	Eugenol	C_10_H_12_O_2_	164	Phenylpropene
8	21.56	4.19	Methyleugenol^*∗*^	C_11_H_14_O_2_	178	Phenylpropene
9	23.47	5.27	4-Hydroxy-2-methoxycinnamaldehyde^*∗*^	C_10_H_10_O_3_	178	Phenylpropene
10	24.55	14.77	Methylconiferylaldehyde^*∗*^	C_11_H_12_O_3_	192	Phenylpropene
11	35.91	7.68	Tetradecanoic acid	C_14_H_28_O_2_	228	Fatty acids
12	26.01	12.04	(E)-2,6-dimethoxy-4-(prop-1-en-1-yl) phenol^*∗*^	C_11_H_14_O_3_	194	Phenylpropene
13	27.97	2.98	Methoxyeugenol^*∗*^	C_11_H_14_O_3_	194	Phenylpropene
14	36.45	1.31	*n*-Hexadecanoic acid	C_16_H_32_O_2_	256	Fatty acids
15	30.03	1.07	Isoelemicin^*∗*^	C_12_H_16_O_3_	208	Phenylpropene
16	34.65	1.77	2-Propenoic acid, 3-(3-methoxyphenyl)-, ethyl ester^*∗*^	C_12_H_14_O_3_	206	Phenylpropene
17	44.52	1.04	Eicosanoic acid^*∗*^	C_20_H_40_O_2_	312	Fatty acids

Nos. 1, 2, 5, 8, 10, 11, and 14 are the same in Tables 3 and 4. ^*∗*^Components that have not been reported in *L. leontopodioides*.

**Table 4:** Chemical composition of *L. leontopodioides* essential oil extracted with diethyl ether.

No.	*t*_*R*_ (min)	Content (%)	Components	Formula	Relative molecular mass	Classification
1	6.23	5.63	Terpinolene∗	C_10_H_16_	136	Monoterpenoids
2	7.11	3.83	*γ*-Terpinene	C_10_H_16_	136	Monoterpenoids
3	8.00	1.79	Allo-ocimene^*∗*^	C_10_H_16_	136	Aliphatic hydrocarbons
4	15.14	2.70	Methyl cinnamate^*∗*^	C_10_H_10_O_2_	162	Phenylpropene
5	10.94	2.43	Terpinen-4-ol	C_10_H_18_O	154	Monoterpenoids
6	19.77	8.63	8-Methylnonanoic acid^*∗*^	C_10_H_20_O_2_	172	Fatty acids
7	23.42	3.98	cis-Methyl isoeugenol^*∗*^	C_11_H_14_O_2_	178	Phenylpropene
8	21.53	1.48	Methyleugenol^*∗*^	C_10_H_10_O_3_	178	Phenylpropene
9	25.93	7.29	Elemicin^*∗*^	C_12_H_16_O_3_	208	Phenylpropene
10	24.48	9.61	Methylconiferylaldehyde^*∗*^	C_11_H_12_O_3_	192	Phenylpropene
11	27.92	3.31	Tetradecanoic acid	C_14_H_28_O_2_	228	Fatty acids
12	35.73	9.25	Pentadecanoic acid	C_15_H_30_O_2_	242	Fatty acids
13	36.40	1.71	Ethyl tridecanoate^*∗*^	C_15_H_30_O_2_	242	Esters
14	44.45	2.12	*n*-Hexadecanoic acid	C_16_H_32_O_2_	256	Fatty acids
15	51.71	5.68	Linoleic acid	C_18_H_32_O_2_	280	Fatty acids
16	51.97	4.06	17-Octadecynoic acid^*∗*^	C_18_H_32_O_2_	280	Fatty acids

Nos. 1, 2, 5, 8, 10, 11, and 14 are the same in Tables 3 and 4. ^*∗*^Components that have not been reported in *L. leontopodioides.*

## Data Availability

The data used to support the findings of this study are included within the article.

## Additional Points

*Highlights*. (1) In this study, we divided the water extract and volatile oil of *L. leontopodioides* to reveal its chemical constituents by UPLC-Q-Orbitrap MS and GC-MS for the first time. In addition, the constituents of volatile oil dissolved in two different solvents were investigated. (2) This is not only a comprehensive and systematic composition analysis of *L. leontopodioides* but also provides a basis for its development and utilization.
